# Chlorthalidone-Induced Fixed-Drug Eruption: Unmasking an Uncommon Reaction to a Common Diuretic

**DOI:** 10.7759/cureus.46199

**Published:** 2023-09-29

**Authors:** Mariana Marrero Castillo, David Kaufman, Juanita Valdes Camacho, Kesler Bourgoyne, John Jacob, Benedict Amalraj, Neerja Gulati

**Affiliations:** 1 Internal Medicine, Ochsner Louisiana State University Health Shreveport - Academic Medical Center, Shreveport, USA; 2 Allergy and Immunology, Ochsner Louisiana State University Health Shreveport - Academic Medical Center, Shreveport, USA; 3 Pulmonary Medicine, Ochsner Louisiana State University Health Shreveport - Academic Medical Center, Shreveport, USA

**Keywords:** fixed-drug eruption, systemic hypertension, hypertension, chlorthalidone, mucocutaneous lesion, thiazide diuretics

## Abstract

Fixed-drug eruptions (FDEs) are dermatological reactions characterized by specific skin lesions triggered by certain medications. Our case reports commonly used medications that can cause drug-induced skin reactions. Chlorthalidone, a widely used diuretic, had not been prominently linked to FDEs. Here, we present the case of a 45-year-old African-American male who developed classic FDE skin lesions following the initiation of chlorthalidone therapy. This case underscores the imperative for further investigation and heightened awareness among healthcare professionals regarding chlorthalidone-associated FDEs. Findings suggest that such reactions might be more prevalent than previously acknowledged, underscoring the significance of prompt diagnosis and effective management of drug-induced skin responses. Notably, the patient's lesions showed complete resolution upon discontinuing the diuretic, reinforcing the causal relationship. This case is an essential reminder of the importance of vigilance in monitoring patients for adverse drug reactions, even in unlikely medications, such as chlorthalidone.​​​​​​

## Introduction

A fixed-drug eruption (FDE) is a skin reaction caused by a specific drug that tends to reappear in the same area(s) where it occurred previously when the same drug is retaken. These skin lesions commonly manifest as one or multiple clearly defined, round patches that appear purplish or reddish [1]. Among the numerous implicated drugs triggering FDE, some include trimethoprim-sulfamethoxazole, analgesics, and anticonvulsants [2,3]​​​​. As an FDE begins to recover, the affected areas develop crusts and scale before gradually changing to a shade of deep purple or dusky brown. These darkened patches can endure and gradually disappear as long as the triggering drug is abstained from. Although typically asymptomatic, FDEs can occasionally manifest with bothersome symptoms, such as pruritus or pain.

## Case presentation

**Figure 1:**
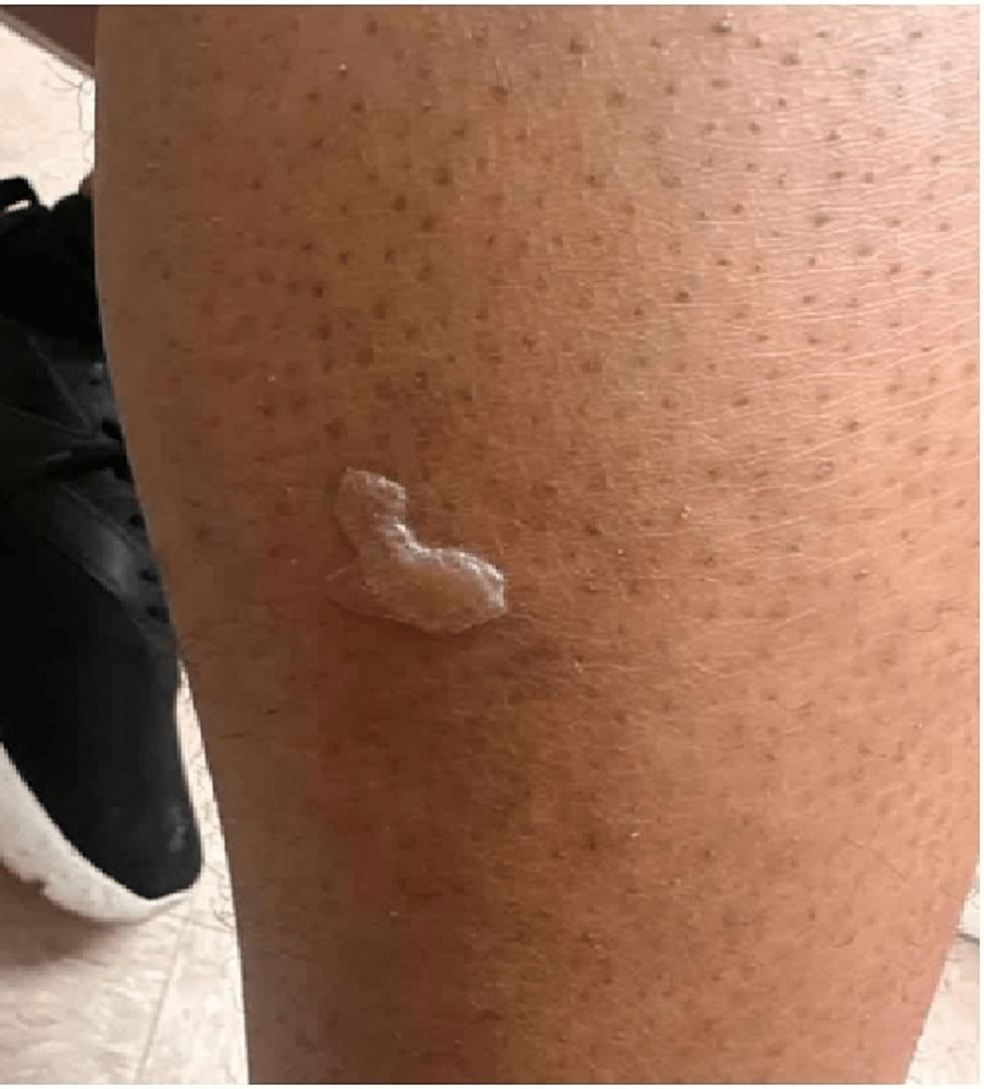
A fluid filled blister on the right leg measuring approximately 3 cm x 3 cm

**Figure 2:**
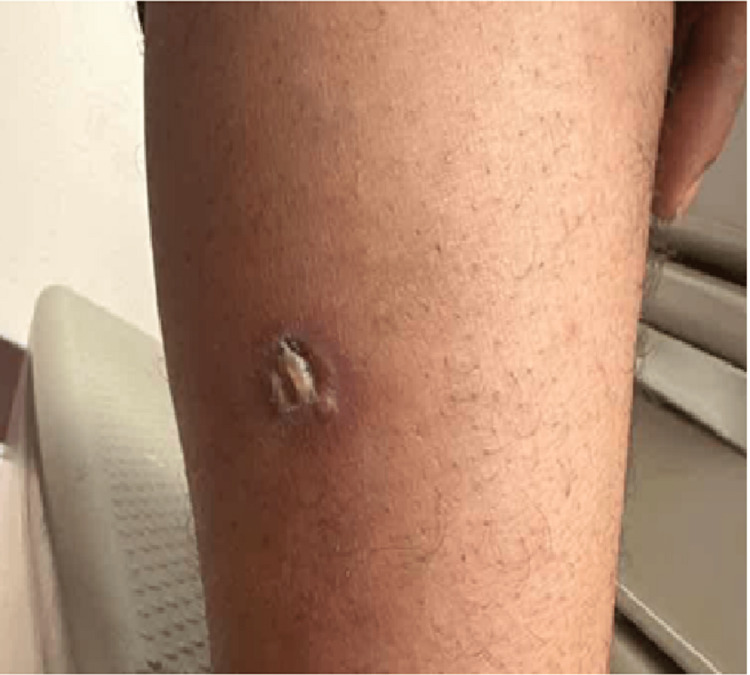
A crusted blister with surrounding erythema on the left leg measuring approximately 3 cm x 3 cm

**Figure 3:**
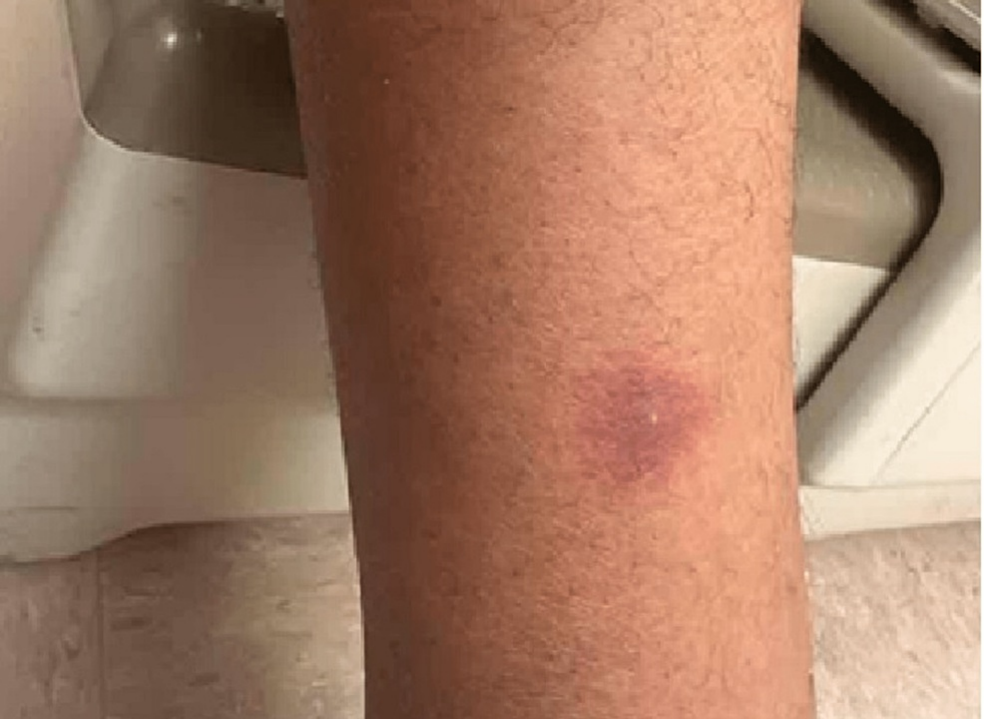
A violaceus non-blanching macule with surrounding erythema on the left leg measuring approximately 2 cm x 2 cm

**Figure 4:**
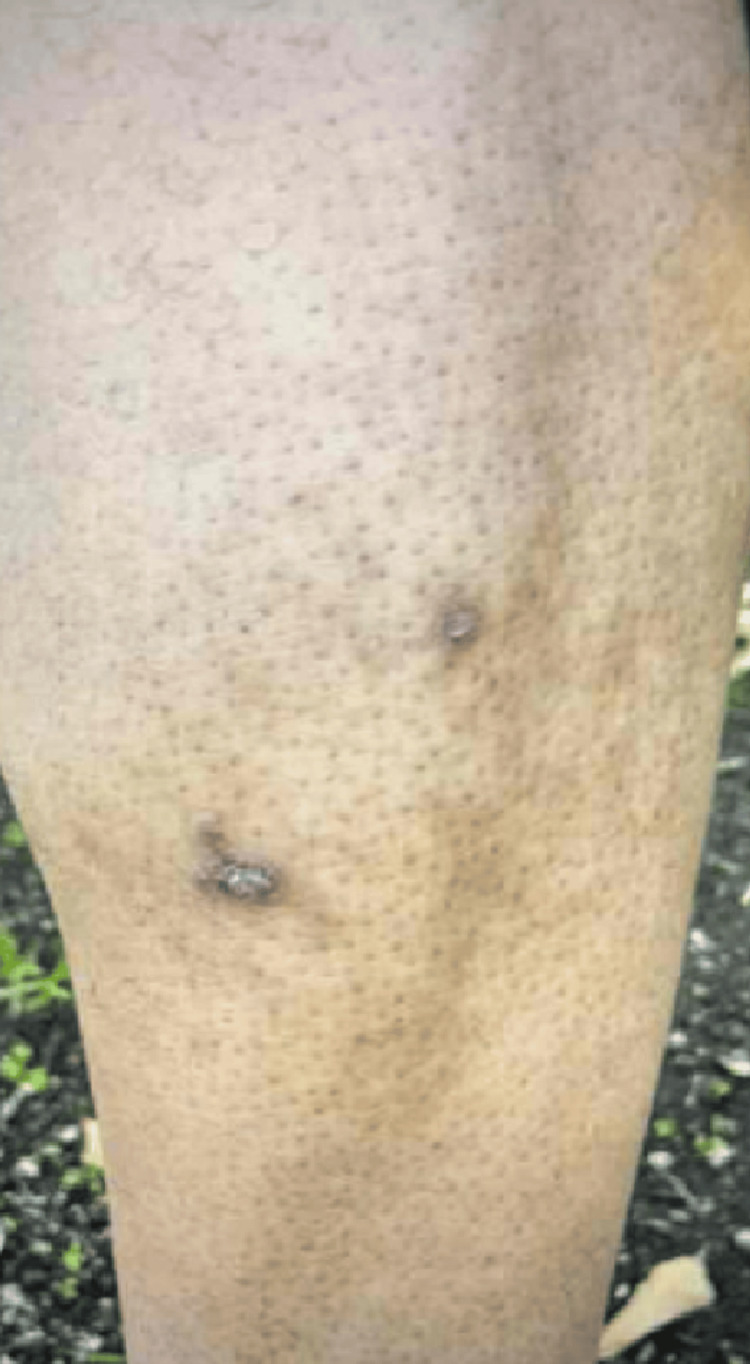
Gradually resolving lesions (left leg) with residual hyperpigmentation

In our case, a 45-year-old African-American male with poorly controlled hypertension on amlodipine presented on December 21, 2022, when chlorthalidone was added to his treatment regimen. However, on December 27, 2022, the patient called the clinic, complaining of multiple new leg lesions. Therefore, the patient was promptly advised to discontinue chlorthalidone and scheduled to visit the internal medicine clinic. The patient was seen by his primary care physician (PCP) on December 28, 2022. Before this, the patient reported no prior history of exposure to thiazide diuretics. During this visit, the patient was started on lisinopril and referred to the allergy and immunology clinic for a more comprehensive evaluation. Images of his skin lesions were captured on this date (December 28, 2022), documenting their presence on the right leg's lateral surface and the left leg's medial surface. There was no involvement of urticarial or oral mucocutaneous regions. The lesions were associated with mild pain and pruritus with no reported excoriations. Physical examination showed a fluid-filled blister on the right leg (measuring 3 cm x 3 cm; Figure 1), a crusted blister on the left leg (3 cm x 3 cm; Figure 2), and a violaceous, non-blanching macule with adjacent erythema on the left leg as well (2 cm x 2 cm; Figure 3). A follow-up visit to the allergy and immunology clinic on January 11, 2023, revealed significant healing of the skin rashes (Figure 4). The patient was recommended to avoid thiazide diuretics permanently.

## Discussion

FDEs are adverse drug reactions that reappear in a similar location with re-exposure to the previous drug. Typically, they manifest as round patches with a reddish-purple hue, although various forms have been documented [1]. The most common presentation of FDEs typically involves circular, hyperpigmented dermal lesions. Less frequent presentations of FDEs may present as non-pigmented erythema, widespread hypermelanosis, urticaria, or periorbital engagement [2].

The defining feature of FDEs is the reappearance of skin eruptions in identical locations when the offending drug is reintroduced, making it a relatively straightforward diagnosis in many cases. In this case, it was suspected that chlorthalidone was the causative agent, as the patient had not been exposed to thiazide diuretics prior to and developed skin lesions shortly after exposure to the medication.

All ages are vulnerable to the development of FDEs. Most cases have been reported in the age range of 20-40 years [3]. The most common drugs causing FDEs are antibiotics (mainly sulfonamides and tetracyclines), analgesics (e.g., ibuprofen and acetylsalicylic acid), antifungals, and antipsychotics [4]. Limited research has investigated the frequency of FDEs associated with chlorthalidone. Some of the antihypertensives that have been described are diltiazem, enalapril, amlodipine, propranolol, atenolol, bisoprolol, nifedipine, hydralazine, and indapamide [5,6,7]​​​​. Some studies showed a trend toward male predominance, although female predominance has also been reported [8].

The numerous clinical and pathologic features observed in FDE lesions are typically explained by intraepidermal CD8(+) T cells with effector memory phenotype in the FDE lesions [9]​​​​. The phenomenon of lesions recurring at the previously involved sites has intrigued many dermatologists. Intraepidermal CD8+ T cells with effector-memory phenotype resident in FDE lesions are important in the disease pathogenesis [10].

Chlorthalidone, a thiazide-type diuretic that inhibits distal convoluted tubule sodium and chloride resorption, is a commonly used oral antihypertensive [11].^ ^This medication is not commonly associated with FDEs, as indicated by the rarity of such cases in the literature. The fact that the patient, in this case, developed FDEs after chlorthalidone exposure suggests that FDEs may be more diverse regarding causative agents than previously thought. This underscores the importance of ongoing research and vigilance among physicians when monitoring patients for adverse drug reactions.

Since FDE diagnosis relies primarily on clinical evaluation, gathering a comprehensive medication history to pinpoint the responsible drug is crucial, as the treatment depends on abstaining from it* *[11,12,13]*.* In cases where diagnosis remains uncertain, patch testing, oral drug challenge, and skin biopsy can serve as valuable diagnostic tools [14]. Not addressing the removal of the causative agent in cases of localized FDEs can result in its return, leading to heightened inflammation, increased pigmentation, and an elevated risk of a potentially life-threatening generalized bullous FDE (GBFDE). GBFDE can resemble to conditions, such as Stevens-Johnson syndrome (SJS) or toxic epidermal necrolysis (TEN) [2].

The decision of lifelong avoidance of thiazide diuretics is significant, as it acknowledges the potential for cross-reactivity between different drugs within the same class, which is a concern for patients with FDEs. Our literature review identified only one prior case in the United States, described as the first documented case [14]. The symptoms typically remit within a few days to weeks upon discontinuation of the implicated medication, although there is a possibility of developing persistent chronic hyperpigmentation [15,16]. 

The case report suggests that chlorthalidone-induced FDEs may be more common than previously expected. While extensive research and systematic reviews have shed light on various drug-induced skin reactions, chlorthalidone, a widely used diuretic, had not been prominently linked to FDEs in prior studies [10,17,18,19]. There are only a few cases in the literature of FDEs secondary to diuretics, such as furosemide or indapamide, and only two cases to chlorthalidone [14,20,21,22].

Another distinctive feature of FDEs, as observed in our case, is that individuals with darker skin tones tend to exhibit more noticeable hyperpigmentation [23]​​​. This highlights the importance of physician awareness of potential adverse drug reactions and the need for a thorough patient history, especially regarding prior exposure to drugs. It also emphasizes the importance of reporting such cases to relevant medical databases to contribute to ongoing research and improve our understanding of drug-related skin reactions.

## Conclusions

FDEs are recognized for their manifestation as skin lesions attributed to specific drugs. Typically, FDEs are associated with a single culpable drug, although combination medications have been implicated. Localized reactions have been associated with key immune cells that play a role in contributing to the cutaneous manifestations of FDEs. The need for continued research and increased awareness among healthcare providers is highlighted, as chlorthalidone-associated FDEs may be more common than previously thought, emphasizing the importance of a timely diagnosis and effective management of drug-induced skin reactions.

This case report highlights the need to better understand chlorthalidone-induced FDEs, shedding light on potential adverse reactions associated with this commonly prescribed hypertension medication. It underscores the vital role of healthcare providers in monitoring patients for adverse drug reactions, especially in cases with atypical presentations or unclear medication histories, to ensure timely diagnosis and intervention, ultimately enhancing patient safety. The report emphasizes the importance of maintaining a high level of suspicion and considering commonly prescribed medications as potential culprits when faced with unusual clinical manifestations, contributing to a safer and more informed approach to drug therapy and improving patient outcomes and healthcare quality.

## References

[1] Shaker G, Mehendale T, De La Rosa C (2022). Fixed drug eruption: an underrecognized cutaneous manifestation of a drug reaction in the primary care setting. Cureus.

[2] Patel S, John AM, Handler MZ, Schwartz RA (2020). Fixed drug eruptions: an update, emphasizing the potentially lethal generalized bullous fixed drug eruption. Am J Clin Dermatol.

[3] Mahboob A, Haroon TS (1998). Drugs causing fixed eruptions: a study of 450 cases. Int J Dermatol.

[4] Ranugha PS, Betkerur JB (2018). Antihypertensives in dermatology part II - cutaneous adverse reactions to antihypertensives. Indian J Dermatol Venereol Leprol.

[5] Zaccaria E, Gualco F, Drago F, Rebora A (2006). Fixed drug eruption due to propranolol. Acta Derm Venereol.

[6] Sehgal VN, Srivastava G (2006). Fixed drug eruption (FDE): changing scenario of incriminating drugs. Int J Dermatol.

[7] Belhadjali H, Trimech O, Youssef M, Elhani I, Zili J (2009). Fixed drug eruption induced by atenolol. Clin Cosmet Investig Dermatol.

[8] S P, K M, S A (2013). Causality, severity and preventability assessment of adverse cutaneous drug reaction: a prospective observational study in a tertiary care hospital. J Clin Diagn Res.

[9] Mizukawa Y, Shiohara T (2009). Fixed drug eruption: a prototypic disorder mediated by effector memory T cells. Curr Allergy Asthma Rep.

[10] Lee C, Chen Y, Cho Y, Chang C, Chu C (2012). Fixed-drug eruption: a retrospective study in a single referral center in northern Taiwan. Dermatol Sin.

[11] Lee AY (2000). Fixed drug eruptions. Incidence, recognition, and avoidance. Am J Clin Dermatol.

[12] Brahimi N, Routier E, Raison-Peyron N (2010). A three-year-analysis of fixed drug eruptions in hospital settings in France. Eur J Dermatol.

[13] Shiohara T (2009). Fixed drug eruption: pathogenesis and diagnostic tests. Curr Opin Allergy Clin Immunol.

[14] Cuervo-Pardo N, Gonzalez-Estrada A, Cuervo-Pardo L, Reddy K, Gonzalez-Estrada A (2018). Bullous fixed drug eruption secondary to chlorthalidone. J Allergy Clin Immunol Pract.

[15] Long CY, Wong N, Burns A (2021). Fixed drug eruption to trimethoprim-sulfamethoxazole and doxycycline. Cureus.

[16] Flowers H, Brodell R, Brents M, Wyatt JP (2014). Fixed drug eruptions: presentation, diagnosis, and management. South Med J.

[17] Pai VV, Kikkeri NN, Athanikar SB, Shukla P, Bhandari P, Rai V (2014). Retrospective analysis of fixed drug eruptions among patients attending a tertiary care center in Southern India. Indian J Dermatol Venereol Leprol.

[18] Jung JW, Cho SH, Kim KH, Min KU, Kang HR (2014). Clinical features of fixed drug eruption at a tertiary hospital in Korea. Allergy Asthma Immunol Res.

[19] heng YK, Yew YW, Lim D, Lim YL (2014). A restrospective review of fixed drug eruptions presenting at a tertiary dermatology centre in Singapore between 2008 and 2012. Clin Transl Allergy.

[20] Rawat R, Joshi Y, Sharma A (2018). Case report on thiazide-type diuretic induced fixed drug eruption. Am J Health Syst Pharm.

[21] Scherschun L, Lee MW, Lim HW (2001). Diltiazem-associated photodistributed hyperpigmentation: a review of 4 cases. Arch Dermatol.

[22] Luhar M, Anusha S, Alias B, Girish H, Ashvil A (2020). Extensive fixed drug eruption due to furosemide: a case report. Int J Pharm Res.

[23] Yim S, Abu-Hilal M (2019). Fixed drug eruption. McMaster Textbook of Internal Medicine.

